# Differentially expressed microRNAs associated with changes of transcript levels in detoxification pathways and DDT-resistance in the *Drosophila melanogaster* strain *91-R*

**DOI:** 10.1371/journal.pone.0196518

**Published:** 2018-04-26

**Authors:** Keon Mook Seong, Brad S. Coates, Do-hyup Kim, Allison K. Hansen, Barry R. Pittendrigh

**Affiliations:** 1 Department of Entomology, Michigan State University, East Lansing, Michigan, United States of America; 2 USDA-ARS, Corn Insects & Crop Genetics Research Unit, Iowa State University, Ames, Iowa, United States of America; 3 Department of Entomology, University of California, Riverside, California, United States of America; Institut de Pharmacologie Moleculaire et Cellulaire, FRANCE

## Abstract

Dichloro-diphenyl-trichloroethane (DDT) resistance among arthropod species is a model for understanding the molecular adaptations in response to insecticide exposures. Previous studies reported that DDT resistance may involve one or multiple detoxification genes, such as cytochrome P450 monooxygenases (P450s), glutathione S-transferases (GSTs), esterases, and ATP binding cassette (ABC) transporters, or changes in the voltage-sensitive sodium channel. However, the possible involvement of microRNAs (miRNAs) in the post-transcriptional regulation of genes associated with DDT resistance in the *Drosophila melanogaster* strain *91-R* remains poorly understood. In this study, the majority of the resulting miRNAs discovered in small RNA libraries from *91-R* and the susceptible control strain, *91-C*, ranged from 16–25 nt, and contained 163 precursors and 256 mature forms of previously-known miRNAs along with 17 putative novel miRNAs. Quantitative analyses predicted the differential expression of ten miRNAs between *91-R* and *91-C*, and, based on Gene Ontology and pathway analysis, these ten miRNAs putatively target transcripts encoding proteins involved in detoxification mechanisms. RT-qPCR validated an inverse correlation between levels of differentially-expressed miRNAs and their putatively targeted transcripts, which implies a role of these miRNAs in the differential regulation of detoxification pathways in *91-R* compared to *91-C*. This study provides evidence associating the differential expression of miRNAs in response to multigenerational DDT selection in *Drosophila melanogaster* and provides important clues for understanding the possible roles of miRNAs in mediating insecticide resistance traits.

## Introduction

While chemical insecticidal agents have been developed and widely applied to suppress pest arthropod populations in ongoing efforts to enhance the efficiency of agricultural production and protect human health [1], this intensive use of chemical insecticides has also led to the development of resistance to one or more classes of insecticides [2–4]. The neurotoxic organochlorine insecticide, dichlorodiphenyltrichloroethane (DDT), has been extensively used to control many agricultural insect pests and insects that vector human diseases, but was banned in most countries in the 1980s due to environmental concerns [5]. The genetic basis of DDT resistance in the *Drosophila melanogaster* (*D*. *melanogaster*) provides a model system for studying the evolution of insecticide resistance. Indeed, the low-level DDT resistance phenotype in *D*. *melanogaster* is thought to be associated with a single cytochrome P450, *Cyp6g1* [6,7]. However, moderate to high-level DDT resistance is polygenic [8] involving modulation in DDT penetrance and excretion and multiple differentially-regulated Phase I, II, and III detoxification genes/enzymes, respectively, including P450s, glutathione S transferases (GSTs), and ATP binding cassette (ABC) transporters. Regarding the P450s, members of the *Cyp6* (*Cyp6g1*, *Cyp6g2*, *Cyp6a2*, and *Cyp6w1*) and *Cyp12* (*Cyp12d1*) subfamilies have been implicated in polygenic DDT resistance [9–13]. Furthermore, the overexpression and structural changes in membrane-spanning ABC transporters were shown to be involved in DDT efflux and contribute to the overall DDT resistance phenotype in *D*. *melanogaster* [14]. Full genome re-sequencing identified 13 different regions showing evidence of high nucleotide diversity, directional selection (selective sweeps), and thus putatively associated with the evolution of DDT resistance in the DDT-resistant strain, *91-R*, when compared with a susceptible isoline, *91-C* [15]. Analyses of this same whole genome re-sequencing data also identified a large panel of fixed amino acid changing mutations between *91-R* and *91-C*, many of which showed evidence of directional selection [16]. Most recently, a multigenic response to DDT selection was demonstrated within *91-R* where transcripts with constitutive differential-expression compared to *91-C* were enriched in cell survival, stress response, and neurological functions [17]. Additionally, reduced expression of the putative calcium/lipid binding domain-containing protein from gene model *CG10737* is associated with short-term (3–5 hrs) DDT knockdown resistance within the *Drosophila* Genetic Reference Panel, suggesting a role for ameliorated effects of muscle overstimulation in DDT resistance phenotypes [13]. Despite these lines of evidence, the systemic basis and underlying genetic control of polygenic DDT resistance remains enigmatic.

Accumulating evidence demonstrates that the post-transcriptional regulation (RNA editing and alternative splicing) may contribute to the evolution of insecticide resistant phenotypes [18,19]. The miRNAs are a class of endogenous 18–25 nt non-coding small RNAs that, since their discovery in *Caenorhabditis elegans* over two decades ago [20], have been identified across several arthropod species including twelve *Drosophila* species [21]. Biogenesis of miRNAs occurs from transcript-derived stem-loop structures with canonical CNNC downstream elements [22] that are processed into a ~70 bp double-stranded precursor RNAs (pre-RNAs) within the nucleus by the ribonuclease type III enzyme Drosha [23]. After translocation to the cytoplasm, miRNA precursors exhibit canonical stem-loop secondary structures, which usually have two arms called the miRNA-5p arm or -3p arm [24]. The pre-RNAs are further processed into mature 21–22 nt miRNAs by Dicer [25,26]. Following the degradation of the passenger strand [27], the resulting single-stranded guide sequence is loaded into the RNA-induced silencing complex (RISC) [28]. Subsequent base pairing of the miRNA guide “seed sequence” (nucleotides 2–7 at the 5' end of guide RNAs) [29] with the 3'-untranslated region (3'-UTR) of target cellular mRNA transcripts most often targets the mRNA for degradation by RISC [30], either blocking translation initiation factor binding or inhibiting elongation factor progression that leads to premature termination [31–33]. Alternatively, a miRNA binding of a target transcript can result in translational up-regulation [34] within their mRNA resulting in inhibition of translation or mRNA degradation [35]. Overall, miRNAs are recognized as potent regulators of eukaryotic gene expression at the post-transcriptional level [36,37] that regulate cell differentiation [38], migration [39], and neuronal development [40]. Approximately 256 *D*. *melanogaster* miRNA precursors have been identified and deposited in the mirBase (Release 21) [24] and have been shown to be involved in regulating biological processes such as development [41], immune response [42], and metabolism [43].

Recent publications implicate a role of specific miRNAs in the regulation of insecticide resistance mechanisms among insect species. Several comparative analyses have estimated significant differences in miRNA expression between chemical insecticide-resistant and -susceptible strains [44,45]. Additionally, miRNAs differentially expressed between insecticidal *Bacillus thuringiensis* (*Bt*) toxin-resistant and -susceptible strains of *Ostrinia furnacalis* were predicted to target potential receptor genes [46]. In *Culex pipiens*, miRNAs differentially expressed between deltamethrin-sensitive and -resistant strains were proposed to mediate the expression of putative cytochrome P450 target genes [47]. Specifically, *Culex pipiens miR-285* and *miR-278* were implicated in pyrethroid resistance through the transcriptional regulation of *Cyp6n23* and *Cyp6ag11* [48,49], as well as a miRNA cluster involved in regulation of *Cyp9j35* and *Cyp325bg3* [50] and upregulation of *miR-932* that regulates transcript levels of the cuticular gene *CpCPR5* [51,52].

To date, the authors have no knowledge of any investigation of the contributions of post-transcriptional gene expression regulation on DDT resistance phenotypes. In order to partially address this knowledge gap, the present study estimated significant quantitative miRNA differences between the highly DDT-resistant *D*. *melanogaster* strain *91-R* compared to the DDT-susceptible strain *91-C*. Additionally, correlation between levels of differentially expressed miRNAs and corresponding putative computationally predicted P450 target transcripts were made within *91-R*. This study provides insights into the role of miRNAs for the regulation of metabolic resistance to DDT as well as the effects of multigenerational DDT selection on the evolution of miRNA-mediated post-transcriptional regulation in a polygenic *D*. *melanogaster* DDT resistance phenotype.

## Materials and methods

### miRNA library preparation, sequencing, and annotation

The DDT-resistant *91-R* and -susceptible *91-C* strains were obtained from Dr. Ranjan Ganguly (University of Tennessee-Knoxville) and developed as described previously [53]. Both strains were reared on a commercially available medium (Jazz-Mix *Drosophila* Food, Fischer Scientific, Cat. No. AS153) under the conditions of 25 ± 1°C, 55–70% relative humidity and a 14 h light /10 h dark cycle. *91-R* has been continually selected by maintaining the flies in colony bottles with the presence of a 150 mg DDT impregnated filter paper disk, while *91-C* was maintained without any exposure to DDT. Recent topical bioassays estimated that *91-R* is ~107-fold more resistant to DDT compared to *91-C* [17].

Three biological replicates of one hundred 3–5 day-old virgin female adults were collected from both *91-R* and *91-C* (*n* = 6). In order to compare the constitutive expression of miRNAs in subsequent analyses (see below), neither population was exposed to DDT within that generation. The small RNAs (sRNAs) were immediately extracted from live collected flies from each replicate using the Qiagen miRNeasy Mini Kit according to the manufacturer’s instructions (Qiagen, Valencia, CA). RNA degradation and contamination were assessed for all samples using an Agilent 2100 Bioanalyzer (Agilent Technologies, Germany), and RNA concentrations were estimated using a NanoDrop One (Thermo, Wilmington, USA). Illumina sRNA libraries were constructed from each pool, and 50-bp single-end (SE50) sequence read data were generated on an Illumina HiSeq 4000 at the Research Technology Support Facility (Michigan State University, East Lancing, MI).

All raw Illumina sequence data were imported into CLC Genomics Workbench v.9.5 (Qiagen) and all reads were processed to remove low-quality reads, poly A sequences, adapters, reads without 3’ adapter, and sequences shorter than 15 nt. Using the “small RNA analysis” tool in CLC Genomics Workbench, annotation of the trimmed read data from each library (*n* = 6) was made by comparing against the known miRNA precursors of *D*. *melanogaster* in the miRBase R.21 (http://www.mirbase.org/; file hairpin.fa); subsequent generation of relative miRNA counts were made within each library. Only tags matching exactly with the mature 5’ or 3’ regions of previously annotated *D*. *melanogaster* miRNAs (miRbase R.21; file mature.fa) were accepted and retained for the further analysis. The sRNA sequencing data with annotated tags were deposited to NCBI Short Read Archive (SRA) with the accession number SRP136631. Additionally, miRDeep2 v.0.0.8 software was used in order to predict novel miRNA candidates [54]. Illumina adapters of sRNA raw sequence reads were trimmed using cutadapt v.1.15 [55]. The trimmed reads were quality-checked and curated using personal perl script. The results were aligned to the *D*. *melanogaster* reference genome (Release 6; dmel-all-chromosome-r6.19.fasta.gz at http://flybase.org/) using bowtie v.1.2.2 [56] and also were mapped to *D*. *melanogaster* non-coding RNA database (dmel-all-tRNA-6.1/8.fasta and dmel-all-miscRNA-6.1/8.fasta file at flybase.org) in order to filter the small conditional RNAs (scRNAs), small nucleolar RNAs (snoRNAs), small nuclear RNAs (snRNAs) and transfer RNAs (tRNAs). Novel miRNAs were then identified against known miRNA precursors (hairpin.fa) and previously annotated miRNAs (mature.fa) using default parameters suggested by the developers [57]. The structure and minimal free energy (MFE) of all potential novel miRNAs were predicted using a RNAfold [58] with algorithms described previously [59]. The MFE ≤ -25 kcal mol^-1^, the randomization test of secondary structure MFE, called randfold *P*-value ≤ 0.05, and miRDeep2 score ≥ 3 were used as the cutoff level to declare them as potential novel miRNAs [60].

### Differential expression of miRNAs

Estimates of miRNA expression level analysis within each replicate library from *91-R* (*n* = 3) and *91-C* (*n* = 3) were performed using the “Annotate and Merge” command on the CLC Genomics Workbench v.9.5 (Qiagen). This procedure used the *D*. *melanogaster* miRBase (release 21, http://www.mirbase.org/) as the primary database and the non-coding RNA database (dmel-all-tRNA-6.1/8.fasta and dmel-all-miscRNA-6.1/8.fasta) as the secondary database for annotation. The read counts of miRNAs were first normalized using the tag per million reads (TPM) method: TPM = (number of mapped reads for each miRNA/total number of mapped reads) ×10^6^. Subsequently, the Empirical analysis of Differential Gene Expression (EDGE) algorithm [61] was used to estimate differences in read counts comparing *91-R* to *91-C* strains, with *P*-values adjusted for multiple testing calculated using the Benjamini–Hochberg false discovery rate (FDR) procedure [62]. The variance in miRNA levels between the *91-R* and *91-C* with a log_2_ fold-change > 1.0 or < -1.0, and a FDR ≤ 0.05, were defined as significant [63].

### Target prediction and functional annotation of differentially expressed miRNAs

The potential target transcripts of miRNAs predicted to be differentially expressed between *91-R* and *91-C* were predicted using three different types of software packages, RNAhybrid [64], DIANA [65], and ComiR [66], using the following criteria: (1) RNAhybrid: the target site prediction was restricted to the 3’-UTR region obtained from the 3’-UTR database of *D*. *melanogaster* (dmel-all-three_prime_UTR-r6.19.fasta at http://www.flybase.org) with MFE ≤ -30 kcal mol^-1^; (2) DIANA: miTG score ≥ 0.8; and (3) ComiR: high threshold ≥ 0.8. Additionally, target gene ontology (GO) and corresponding pathways for all putative target transcripts were retrieved from the FlyMine database (http://www.flymine.org), and the GO terms were classified with CateGOrizer (https://www.animalgenome.org).

### Validation and correlated expression between miRNAs and target transcripts

Reverse transcriptase-quantitative PCR **(**RT-qPCR) was carried out on selected eight known miRNAs for validation of sRNA sequencing estimated differences between the *91-R* and *91-C* strains. Moreover, the correlation was assessed between the relative expression levels of eight differentially expressed miRNAs and their potential targeted detoxification genes: *Cyp6a8*, *Cyp6g1*, *Cyp6w1*, *Cyp4s3*, *Cyp6g2*, *Cyp309a2*, *Cyp313a4*, *Cyp313b1*, *Cyp4ae1*, *Cyp4d2*, *Cyp4g15*, *Cyp4p3*, *Cyp6d5*, *Cyp6t3*, *Cyp6u1*, *Cyp6v1*, *Cyp49a1*, *Cyp18a1*, *Cyp303a1*, *Cyp4aa1*, *Cyp4e3*, *Cyp4g1*, *Cyp6a19*, *Cyp6a22*, *Cyp6a9*, *Cyp4d20*, *GstD1*, *GstS1*, *GstE6*, *GstE10*, *Esterase10*, *Esterase7*, *Esterase8*, *ABC-B7*, *GABA-R*, *mAChR-A*, *GluR-IB*, *nAcRalpha-A*, and *Cpr65Ec*. Three biological replicates of 15 adult female flies were sampled per strain (*91-R* and *91-C*) and—identically with treatments used in Illumina sRNA sequencing—all samples were not exposed to DDT within the generation used. Both miRNAs and total RNAs were extracted from each biological replicate using a miRNeasy Mini Kit and a RNeasy Mini Kit, with resulting extracts treated with DNAse I (Qiagen) to remove contaminating genomic DNA. The first-strand cDNA was synthesized from mature miRNA and total RNA respectively with a miScript II RT kit (Qiagen) and a SuperScript™ III Reverse Transcriptase kit (Invitrogen, Carlsbad, CA) according to the manufacturer’s instructions. RT-qPCR reactions were performed with a miScript SYBR^®^ Green PCR Kit (Biorad, Hercules, CA) according to the manufacturer’s instructions using miRNA-specific forward primers (S1 Table) with miScript II RT kit (Qiagen) products as a template. Analogously, RT-qPCR reactions for corresponding putative target mRNA transcripts were performed using SYBR^®^ Green Master Mix (Biorad) according to the manufacturer’s instructions using target transcript-specific forward and reverse primers (S1 Table), with products from the SuperScript™ III Reverse Transcriptase kit used as a template. All amplification reactions were performed on a StepOnePlus Real-Time PCR system (Applied Biosystems Inc., Foster City, CA), with three technical replicates across all biological replicates. Normalized miRNA and target transcript expression levels were calculated using the 2^−ΔΔC(t)^ method [67] with the internal references *U6 snRNA* and *5S rRNA* for miRNA normalization and *rp49* for mRNA normalization, respectively. A one-way ANOVA was used to examine the significance of expression differences between the two samples using XLSTAT software (Addinsoft, USA). The correlation coefficient was determined by Pearson's correlation analysis between the transcript levels of the selected eight miRNAs and their corresponding putative target genes.

## Results

### miRNA library preparation, sequencing, and annotation

Six miRNA libraries (three biological replicates from each of *91-R* and *91-C* strains) were constructed, from which sequencing generated 216.2 million total raw reads (≥ 30.1 million per library; Table 1). After trimming reads (i.e., removal of reads without a 3' adaptor and < 15 nt) 5.1 to 5.8 and 4.4 to 7.7 million reads were retained respectively among triplicates from *91-R* and *91-C* (Table 1). These were then used in subsequent analyses. Considering trimmed reads across all six libraries, the majority of the sequences (55.5%) were distributed from 16–25 nt (S1 Fig), which is the standard size of described miRNAs [68]. A class of 26–31 nt long RNA sequences accounted for 25.4% of the total reads (34,598,261) and were classified as suspected piwi-interacting RNAs (piRNAs) [69]. Of the trimmed reads, 531,843 to 992,816 miRNA tags were identified across all replicate libraries of *91-R* and *91-C (*Table 1), and among these between 6,050 and 9,356 matched *D*. *melanogaster* miRNA entries in the miRBase R.21 database.

**Table 1:** Small RNA sequences from *91-C* and *91-R* triplicate libraries read data.

**Category**	**Analyses of total reads data**					
	***91-R*-1**	***91-R*-2**	***91-R*-3**	***91-C*-1**	***91-C*-2**	***91-C*-3**
Raw reads	35,712,203	30,108,348	33,996,366	43,821,999	34,205,253	38,365,489
Trimmed reads	5,801,176	5,105,542	5,439,260	7,738,822	6,065,134	4,448,327
Annotated with ncRNA (rRNA, tRNA, snRNA, snoRNA and others)	4,399,524	4,072,155	4,196,227	5,961,313	4,553,746	3,182,916
*D*. *melanogaster* miRNAs	384,620	252,554	339,001	455,995	336,210	356,146
Unannotated	1,017,032	780,833	904,032	1,321,514	1,175,178	909,265
**Data Processing/** **Strains**	**Analyses of unique reads**					
	***91-R*-1**	***91-R*-2**	***91-R*-3**	***91-C*-1**	***91-C*-2**	***91-C*-3**
Unique miRNA	992,816	920,013	944,922	900,308	894,908	531,843
Annotated with ncRNA (rRNA, tRNA, snRNA, snoRNA and others)	490,904	510,823	503,922	344,222	375,672	174,207
*D*. *melanogaster* miRNAs	7,643	6,050	7,079	9,356	7,947	7,942
Unannotated	494,269	403,140	433,921	546,730	511,289	349,694

Annotation of known miRNAs in the *91-R* and *91-C* strains identified 163 precursor and 256 mature miRNAs following alignment of trimmed reads to the *D*. *melanogaster* precursor/mature miRNAs in the miRBase R.21 (S2 Table). None of the precursor or mature miRNAs were expressed uniquely in either strain. However, we failed to identify any of the 93 known *D*. *melanogaster* precursors that are recorded in the miRBase R.21 (S3 Table). In both strains, *miR-184-3p* miRNA was the most abundant among the means of non-normalized reads across triplicate libraries, with *miR-8-3p*, *miR-276a-3p*, *bantam-3p*, and *miR-33-5p* as the next most abundant miRNAs in both strains (Table 2). Algorithms in the miRDeep2 package identified 17 potential novel miRNAs in *91-R* and *91-C*, of which 15 were in common between libraries derived from both strains; putatively *novel-miR-3L-18860981* and *novel-miR-2R-20583765* were uniquely observed in *91-R* and *91-C*, respectively (S4 Table). The range of estimated MFE among potential novel miRNAs was between -31.7 and -25.5 kcal mol^-1^ and their mature lengths from 18–25 nt. These 17 novel miRNAs were named based on the chromosome and position on which the miRNA gene is located in the *D*. *melanogaster* genome.

**Table 2:** The most abundant reads from *91-R* and *91-C* small RNA libraries corresponding to known *Drosophila melanogaster* miRNAs in miRBase R.21.

miRNA	*91-R* Read counts^a^	*91-C* Read counts^a^	Mature sequence
*miR-184-3p*	27,954	27,402	UGGACGGAGAACUGAUAAGGGC
*miR-8-3p*	24,289	25,956	UAAUACUGUCAGGUAAAGAUGUC
*miR-276a-3p*	20,174	17,849	UAGGAACUUCAUACCGUGCUCU
*bantam-3p*	7,498	9,036	UGAGAUCAUUUUGAAAGCUGAUU
*miR-33-5p*	4,208	5,040	GUGCAUUGUAGUCGCAUUGUC
*miR-10-5p*	3,464	3,954	ACCCUGUAGAUCCGAAUUUGUU
*miR-317-3p*	2,908	3,292	UGAACACAGCUGGUGGUAUCCAGU
*miR-14-3p*	2,603	2,611	UCAGUCUUUUUCUCUCUCCUAU
*miR-31a-5p*	3,580	2,354	UGGCAAGAUGUCGGCAUAGCUGA
*miR-312-3p*	1,056	2,318	UAUUGCACUUGAGACGGCCUGA
*miR-11-3p*	1,659	2,275	CAUCACAGUCUGAGUUCUUGC
*miR-311-3p*	596	2,170	UAUUGCACAUUCACCGGCCUGA
*miR-318-3p*	994	1,977	UCACUGGGCUUUGUUUAUCUCA
*miR-999-3p*	1,604	1,660	UGUUAACUGUAAGACUGUGUCU
*miR-957-3p*	1,744	1,576	UGAAACCGUCCAAAACUGAGGC
*miR-276b-3p*	1,846	1,553	UAGGAACUUAAUACCGUGCUCU
*miR-277-3p*	2,046	1,489	UAAAUGCACUAUCUGGUACGACA
*miR-995-3p*	542	1,275	UAGCACCACAUGAUUCGGCUU
*miR-305-3p*	1,000	1,235	CGGCACAUGUUGAAGUACACUCA
*let-7-5p*	1,094	1,208	UGAGGUAGUAGGUUGUAUAGU
*miR-986-5p*	3,069	139	UCUCGAAUAGCGUUGUGACUGA
*miR-958-3p*	1,020	899	UGAGAUUCUUCUAUUCUACUUU

^a^ Non-normalized reads summed across triplicate libraries

### Differential expression analysis and RT-qPCR validation

Comparison of normalized estimates of miRNA quantity (log_2_ fold-changes; S5 Table) demonstrated an overall parity across replicated libraries derived from DDT resistance strain, *91-R*, as compared to the susceptible control, *91-C* (counts pooled across replicates within strain; S2 Fig). Exceptions were found among ten known miRNAs that showed significant levels of differential expression between *91-R* and *91-C* (log_2_ fold-change ≥ |1|; FDR ≤ 0.05). Specifically, nine miRNAs were significantly down-regulated, and only one (*miR-986-5p*) was significantly up-regulated in *91-R* (25.3-fold) when compared with the susceptible control *91-C* (Table 3). Interestingly, four out of these nine down-regulated miRNAs [*miR-310*, *miR-311*, *miR-312* and *miR-313*] are clustered miRNAs and belong to the miR-310 family. Overall, most of the differentially expressed miRNAs were down-regulated in the DDT resistant strain *91-R*, suggesting that those down-regulated miRNAs may be potentially involved in DDT resistance.

**Table 3:** Differentially expressed miRNAs between *91-C* and *91-R*.

miRNA	*p*-value	FDR^a^	Fold-change	Log_2_ fold-change^b^	miRNA sequence
*miR-986-5p*	3.0E-53	7.8E-51	25.3	4.66	TCTCGAATAGCGTTGTGACTGA
*miR-2a-1-3p//* *miR-2a-2-3p*	7.0E-04	1.3E-02	-2.01	-1.01	TATCACAGCCAGCTTTGATGAGC
*miR-312-3p*	1.4E-07	5.3E-06	-2.02	-1.01	TATTGCACTTGAGACGGCCTGA
*miR-995-3p*	2.8E-09	1.4E-07	-2.17	-1.12	TAGCACCACATGATTCGGCTT
*miR-286-3p*	2.1E-03	3.2E-02	-2.29	-1.19	TGACTAGACCGAACACTCGTGCT
*miR-92a-3p*	2.9E-10	1.9E-08	-2.34	-1.23	CATTGCACTTGTCCCGGCCTAT
*miR-4919-3p*	1.4E-04	3.2E-03	-2.57	-1.36	TAATCCCTGAACGACTTGCAG
*miR-311-3p*	1.8E-15	1.5E-13	-3.38	-1.76	TATTGCACATTCACCGGCCTGA
*miR-310-3p*	9.1E-16	1.2E-13	-3.94	-1.98	TATTGCACACTTCCCGGCCTTT
*miR-313-3p*	4.4E-07	1.4E-05	-4.89	-2.29	TATTGCACTTTTCACAGCCCGA

^a^ FDR: False discovery rate. Differentially expressed miRNAs were identified at the threshold [FDR < 0.05 and log_2_(fold change) ≥|1.0|] of *91-C*/*91-R*.

^b^ Fold change was calculated as *91-C*/*91-R*.

**Fig 1:**
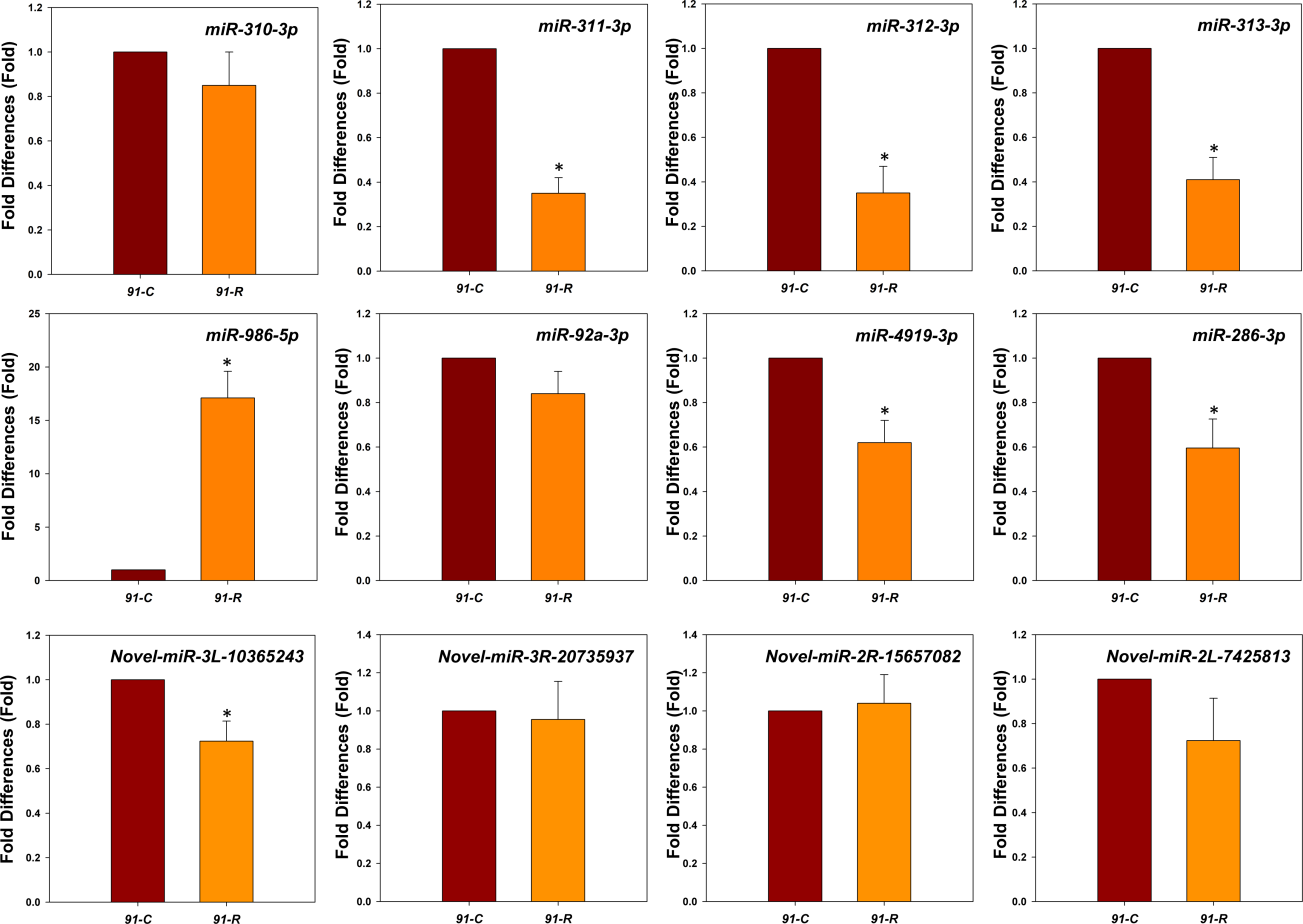
RT-qPCR validation of differentially expressed known and novel miRNAs identified between *91-R* and *91-C* strains. The expression levels were normalized by both *U6* and *5S rRNA*. Statistical significance was analyzed using one-way ANOVA. The asterisks represent significance, where one asterisk indicates *P* < 0.05.

The RT-qPCR validation of the predicted significant quantitative differences in miRNA levels among eight known and four novel miRNAs were amplified showing that the expression levels of *miR-986-5p* were highly up-regulated in *91-R*, whereas *miR-286-3p*, *miR-4919-3p*, *miR-311-3p*, *miR-312-3p*, and *miR-313-3p* were significantly down-regulated in *91-R* (Fig 1). However, levels of *miR-92a-3p* and *miR-310-3p* showed no significant difference between strains (*P*-value > 0.05). Based on Pearson’s correlation coefficient test, the expression patterns of selected miRNAs showed a similar trend between the results of sRNA sequencing and RT-qPCR (R^2^ = 0.971, *P*-value < 0.01), confirming the predicted differential expression between strains. Additionally, the expression level of one putative novel miRNA (*novel-miR-3L-10365243*) was significantly down-regulated in *91-R*, whereas three other putative novel miRNAs showed no significant differences between two strains (Fig 1).

### Target transcript predictions and correlated expression between miRNAs and target transcripts

**Fig 2:**
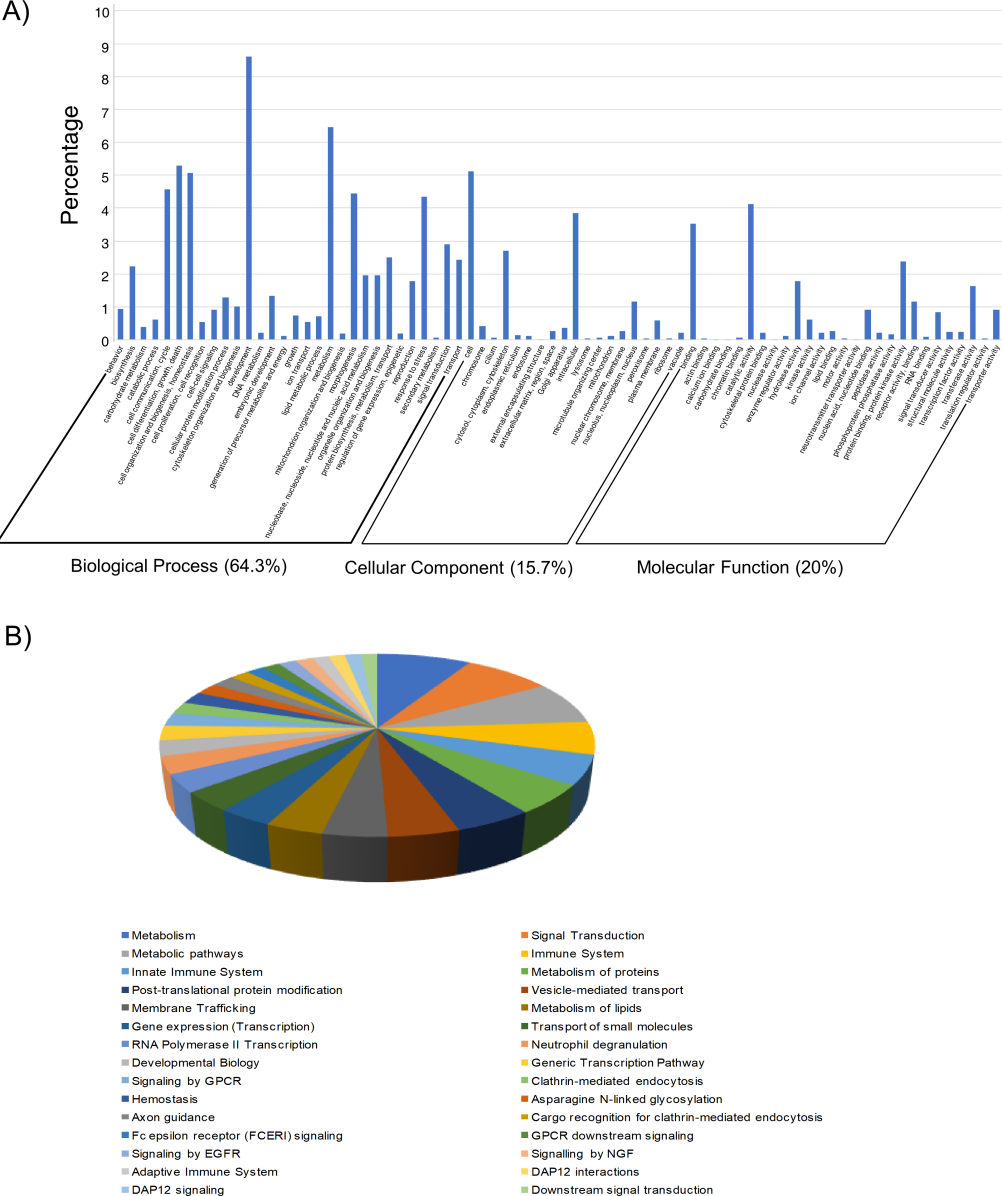
Gene ontology (GO) analysis and pathway annotation of the most targeted genes of the known differentially expressed miRNAs between *91-R* and *91-C* strains. (A) GO analysis. 664 target genes from miRNAs predicted to be differentially expressed were analyzed with FlyMine to obtain the GO terms, and the GO terms were classified with CateGOrizer and separated into three major categories. (B) Pathway annotations. 664 target genes from miRNAs predicted to be differentially expressed were submitted to FlyMine to get the pathway classification. The 30 most abundant pathways were represented.

Considering only the ten miRNAs predicted to be differentially expressed between *91-R* and *91-*C, a total of 46,368 miRNA-target pairs were predicted by the algorithms applied by ComiR (*n* = 5,577), DIANA (*n* = 2,548), and RNAhybrid (*n* = 38,243; S6 Table). Overlap in output occurred among 664 transcript targets predicted by all three algorithms. Functional GO annotations were received for 603 of these 664 transcript targets, with 64.3%, 20%, and 15.7% that respectively received terms in biological process (BP), molecular function (MF), and cellular component (CC) categories (GO level 2; S7 Table). Specifically, two putative functions (development and metabolism) were highly represented in the BP category, and the MF category showed the function of ‘binding’ and ‘catalytic activity’ was most prevalent among target genes. Moreover, cell, intracellular component, cytosol, cytoplasm, and cytoskeleton were largely overrepresented in CC category (Fig 2A). A total of 2,175 biological pathways were assigned to 258 of the 664 predicted transcript targets (38.9%; Fig 2B; S8 Table), and the target *Ras85D* (CG9375) is associated with regulation of tissue growth and development represented 93 biological pathways. The remaining 405 transcript targets (61.1%) received no pathway annotations. Among these 2,175 biological pathways, 58 (2.7%), 54 (2.5%), and 53 (2.4%) target genes of the known differentially expressed miRNAs, respectively, were assigned to metabolism, signal transduction, and metabolic pathway (Fig 2B).

**Fig 3:**
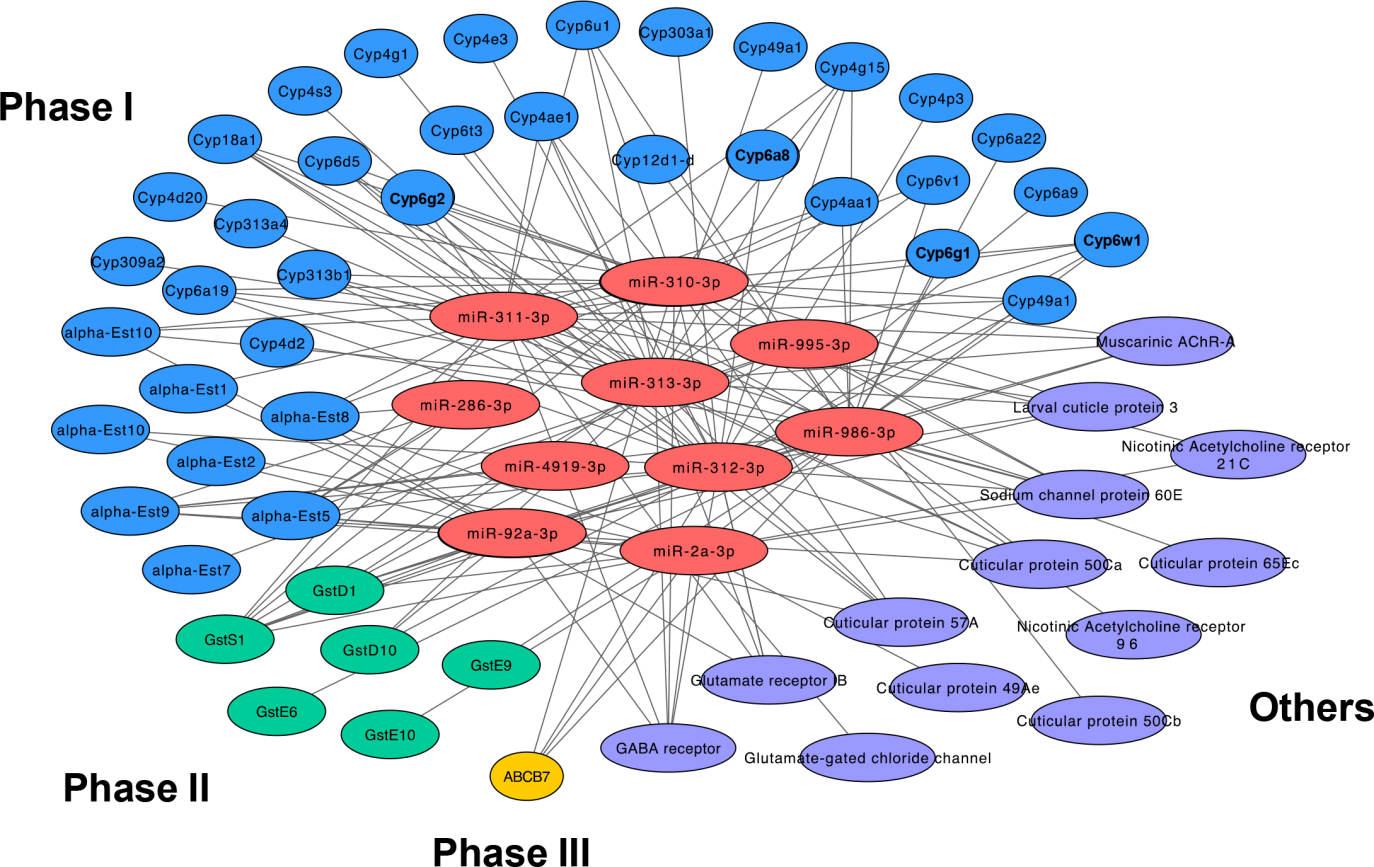
Putative xenobiotic metabolism-related target genes of differentially expressed miRNAs. The network consists of differentially expressed miRNAs and their corresponding target genes. Red hexagons represent differentially expressed miRNAs; blue circles represent target genes as phase I detoxification genes; green circles represent target genes encoding phase II detoxification genes; yellow circle represents target genes as phase III detoxification gene; and purple circles represent other genes affecting the insecticide insensitivity in insects. The network was generated and visualized in Cytoscape v3.6.0. *Cyp6a8*, *Cyp6g1*, *Cyp6g2*, and *Cyp6w1* associated with DDT resistance are in bold.

Additional annotation focused on a subset of the 664 transcripts putatively targeted by differentially expressed miRNAs; specifically, those transcripts likely to be involved in xenobiotic metabolism. These putative transcripts were predicted to target phase I, II, and III detoxification pathways such as cytochrome P450s, GSTs, esterases, and ABC transporters (43 target transcripts; Fig 3). Additionally, differentially expressed miRNAs were predicted to regulate other genes associated with cuticular proteins, acetylcholine receptors, nicotinic acetylcholine receptors, and glutamate-gated chloride channels (13 target transcripts; Fig 3). The phase I, cytochrome P450 targets *Cyp6a8*, *Cyp6g1*, *Cyp6g2*, and *Cyp6w1*, were previously associated with DDT resistance as described in the Introduction. Also of note, the down-regulated *miR-310-313* cluster in *91-R* strain was predicted to share several P450s, sodium channel proteins, and cuticular proteins encoding target genes (Fig 3). Additionally, sixteen P450 genes (MFE ≤ -25; *Cyp18a1*, *Cyp305a1*, *Cyp309a2*, *Cyp312a1*, *Cyp313b1*, *Cyp49a1*, *Cyp4ae1*, *Cyp4g1*, *Cyp4g15*, *Cyp4p2*, *Cyp4s3*, *Cyp6a19*, *Cyp6g1*, *Cyp6g2*, *Cyp6v1*, and *Cyp9h1*) were among the predicted targets of 9 of the 17 putative novel miRNAs (S9 Table).

**Fig 4:**
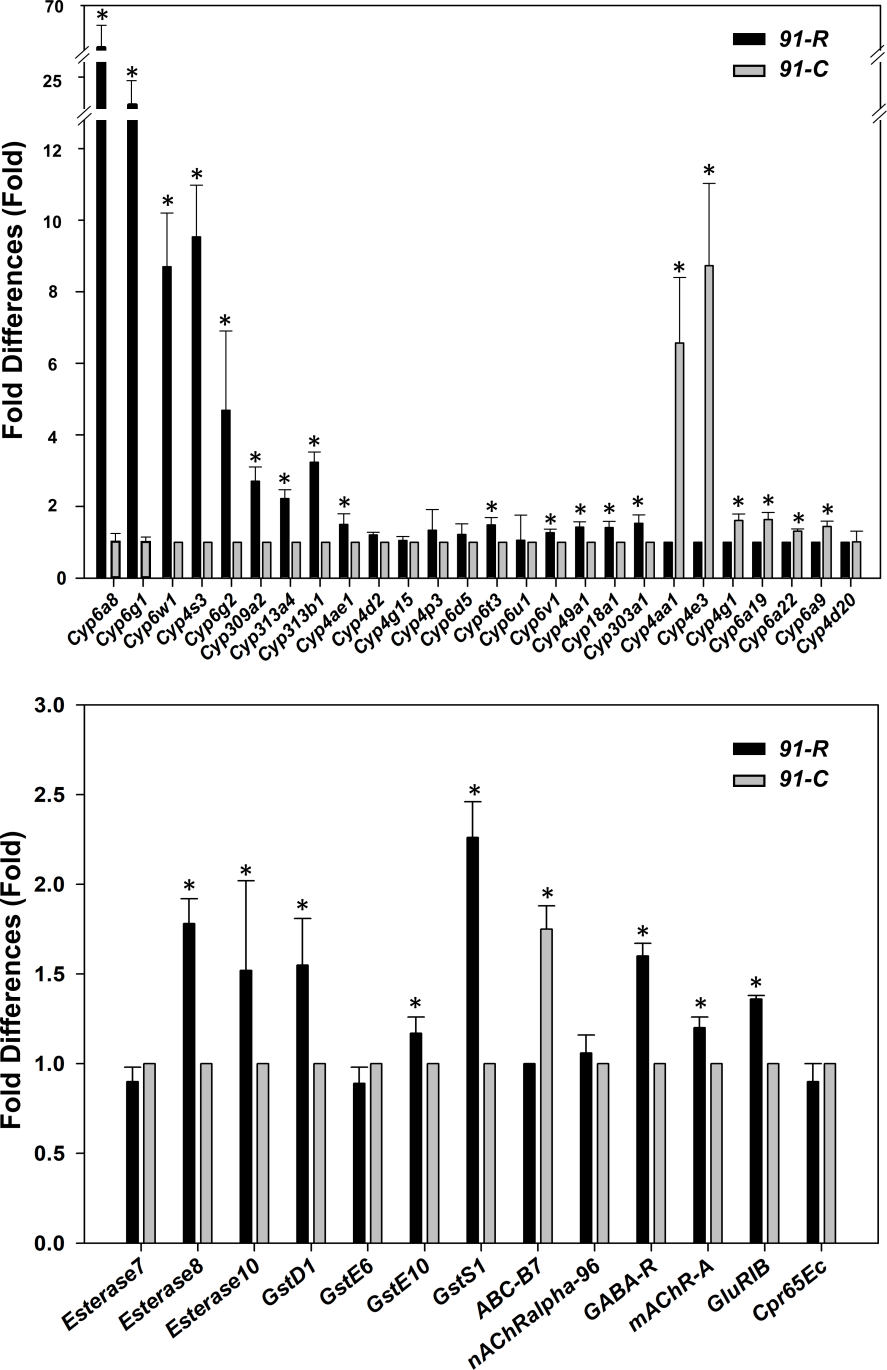
Expression level analysis of potential target detoxification genes of differentially expressed miRNAs between *91-R* and *91-C* strains. The expression levels were normalized by *rp49*. Statistical significance was analyzed using one-way ANOVA. The asterisks represent significance, with a single asterisk indicating *P* < 0.05.

Moreover, the RT-qPCR-estimated quantities of 26 putatively targeted P450 transcripts showed an inverse relationship with the expression level of the corresponding miRNA(s) predicted to target them in *91-R*. For example, the relative expression of three miRNAs (*miR-311-3p*, *miR-312-3p*, and *miR-313-3p*) were significantly down-regulated in *91-R* strain (Fig 1), while the corresponding predicted targets (*Cyp6a8*, *Cyp4s3*, *Cyp4ae1*, *Cyp6g1*, *Cyp6g2*, *Cyp6t3*, *Cyp6v1*, *Cyp18a1*, *Cyp49a1*, *Cyp303a1*, *Cyp309a2*, *Cyp313a4*, and *Cyp313b1*) were significantly up-regulated (Fig 4). Furthermore, other corresponding predicted detoxification targets, *Esterase8*, *Esterase10*, *GstD1*, *GstE10*, *GstS1*, *GABA-R*, *mAChR-A*, *nAcRalpha-A* and *GluR-IB* were significantly up-regulated with the down-regulation of *miR-286-3p*, *miR-2a-3p*, *miR-311-3p*, *miR-312-3p*, and *miR-313-3p* in *91-R* strain (Fig 4). The resulting coefficient of correlation showed that the strong negative correlation between miRNAs and putative target transcripts (R = -0.9558; *P*-value < 0.01) suggests the possibility that higher levels of detoxification transcripts may be influenced by the reduced levels of corresponding targeting miRNAs within the DDT resistant *91-R* strain. The precise role of these detoxification genes in mediating DDT resistance remains unknown, but may include increasing cellular resistance to oxidative stress, cuticular penetrance, or other systemic responses to exposure.

## Discussion

Resistant phenotypes have evolved within arthropod populations that lead to survival when exposed to various chemical or biological insecticidal toxins and seemingly arise to each successively introduced novel mode of action [70]. Although direct correlations have been made between single transposon-based mutations at single genetic loci (an *Accord* element associated with *Cyp6g1*) and corresponding insecticide resistance traits [71–73], in many instances the causal mutations and molecular mechanisms involved in resistance are yet to be fully understood. Moreover, definitive linkages between phenotypes and corresponding adaptive mutations remain difficult to define [74], especially in instances where phenotypes arise due to the contribution of multiple genes or gene interactions, or are complexed with variance due to the environment [75]. For instance, DDT resistance was linked to the up-regulation of *Cyp6g1* caused by the upstream integration of an *Accord* transposon at the DDT-R locus in *D*. *melanogaster* populations [71] but shown to be independent of DDT-R in the highly DDT resistant strain *91-R* [76]. The prediction of thirteen selective sweeps that became fixed in the genome of *91-R* during the course of DDT selection implicated a complex polygenic mode of evolution [15], which likely involves a combination of *cis*- and *trans*-regulatory mutations that modulate the function of stress response and neurogenic pathways [17]. Regardless of evidence that strongly implicates miRNAs as potent modulators of gene expression at the post-transcriptional level [77], the role of miRNAs in the evolution of differential gene expression in insecticide resistant phenotypes among arthropods remains suggestive or associative in many cases [44,45].

The current study identified a total of 163 precursors with 256 mature known miRNAs and 17 novel miRNAs. This study failed to identify 93 known precursor miRNAs, previously recorded in the miRBase R.21. The extraction of sRNAs from 3–5 day-old virgin adult female samples may likely have biased the number of miRNA types. Previously, miRNAs were shown to be sex-biased in *D*. *melanogaster*, where expression was preferentially associated with the reproductive functions [78]. More recent work that compared miRNA expression between mature male and female reproductive organs in *Bactrocera dorsalis* demonstrated that sex-biased miRNAs are likely involved in sexual differentiation [79]. Furthermore, the expression of miRNAs varied across developmental stages of *Xenopus laevis* [80] and across different human tissues [81]. This suggests that our use of virgin female adults might have narrowed the pool of potential miRNAs that could be encountered within the resultant sRNA libraries but is justified since adults are exposed to DDT selection within the *91-R* laboratory colony. Another hypothesis may reside in the potential saturation of sRNA samples with 2S ribosomal RNA (rRNA). Specifically, *D*. *melanogaster* rRNA is composed of four individual rRNAs, 28S, 18S, 5.8S, and 2S, with the 2S rRNA being 30-nt in length [82]. Analysis of our sRNA read data revealed that 32% of non-miRNA sequences matched the *D*. *melanogaster* 2S rRNA, suggesting the possibility that the 2S rRNA component of our libraries might have affected the subsequent read depths and the inability to identify 93 known miRNAs from *91-R* and *91-C* strains if they would have been at low copy number. Neither of the above explanations can be ruled out but require additional investigation. The current study nonetheless predicted the significant quantitative difference in ten miRNA levels between *91-R* and *91-C* at the adult virgin female stage.

Ten differentially expressed miRNAs and their corresponding putative target transcripts were predicted and received GO and pathway database annotations in this study. Among these ten differentially expressed miRNAs, four members of the miR-310 family, *miR-310*, *miR-311*, *miR-312* and *miR-313*, were significantly down-regulated in *91-R* when compared with the *91-C* strain. The miR-310 family form a cluster on chromosomal arm 2R between the CDS of *qsm* and *Nnf1a* (positions 20,583,752 to 20,584,260) [83] and arose via duplication from the ancestral *miR-91* [84]. Previous studies showed that the miR-310 family regulates genes in the *D*. *melanogaster* Toll-mediated innate immune response pathway [85], via hedgehog signaling [86], and beta-catenin that in turn regulates cell adhesion and outgrowth [87]. Furthermore, the *mir-310-313* cluster has been reported to be associated with hypersensitivity to nicotine in *D*. *melanogaster* [88]. These authors observed that the expression of the *miR-310-313* downregulates *escargot* (*esg*) gene expression involved in the development of sensory organs and neurons in the thoracoabdominal ganglion. The overexpression of the *miR-310-313* induces an abnormal sensitivity to nicotine by abating *esg* transcription, then disrupting sensory organs involved in chemical perception and cuticle development. This mechanism may be similar to the DDT resistance of miR-310 family members in *91-R* strain. Additionally, miR-310 family members impact synaptic functions through the regulation of neurotransmitter release at neuromuscular junctions of *D*. *melanogaster* larvae [89]. These authors observed that *miR-310-313* cluster regulates neurotransmitter release at presynaptic terminals by decreasing the expression of *Kinesin heavy chain-73*, *Khc-73*, which is involved in early neural development. Interestingly, our prior research validated the significant differential regulation of genes directing neuronal development in *91-R*, including *Unc-115b*, and *CG31832* that function in neural growth and development, but detected no significant difference in expression of *Khc-73* [17]. Our prior results suggest the possibility that adaptive responses to multigenerational neurotoxic DDT selection in *91-R* may affect the function of neuromuscular junctions, although the role of the miR-310 family in the regulation of genes associated with neuronal functions in *91-R* remains a hypothesis that needs to be tested experimentally.

Findings from this study show that the down-regulation of *miR-311-3p*, *miR-312-3p*, and *miR-313-3p* is correlated with the up-regulation of their respective *in silico* predicted cytochrome P450 target transcripts, *Cyp6a8*, *Cyp4s3*, *Cyp4ae1*, *Cyp6g1*, *Cyp6g2*, *Cyp6t3*, *Cyp6v1*, *Cyp18a1*, *Cyp49a1*, *Cyp303a1*, *Cyp309a2*, *Cyp313a4*, and *Cyp313b1* in *91-R*. This up-regulation of *Cyp6g1* target is predicted to occur via a decreased *miR-310-313* expression in *91-R* and also corresponds to the cytochrome P450 initially implicated in conferring DDT resistance at the DDT-R locus via integration of the *Accord* transposon among *D*. *melanogaster* populations [71]. Our computational and empirical data suggests that the miR-310 family members may be involved in the posttranscriptional regulation of these cytochrome P450s, which in turn might be directly involved in DDT detoxification or stress response [17]. Thus, the involvement of *miR-310-313* could explain prior evidence that even though DDT resistance in *91-R* is independent of the *Accord* insertion [76], significant up-regulation of *Cyp6g1*, as well as *Cyp6a8*, *Cyp4s3*, *Cyp4ae1*, Cyp6g2, *Cyp6t3*, *Cyp6v1*, *Cyp18a1*, *Cyp49a1*, *Cyp303a1*, *Cyp309a2*, *Cyp313a4*, and *Cyp313b1* occurs constitutively in the strain. Regardless of these strong correlations, additional functional analyses are required to confirm these predicted impacts of miRNA-based posttranscriptional regulation.

The function of the most highly up-regulated miRNA in *91-R* strain, *miR-986-5p*, is currently unknown, but levels in the hemolymph of adult virgin males are known to significantly decrease over time [90]. Interestingly, the *miR-986* precursor is located on the second chromosome within the third intron of the *Cyp4e2* gene, which is involved in the metabolism of endogenous and exogenous compounds [91]. Our transcript target site predictions suggest that *miR-986-5p* could interact with transcripts of cytochrome P450s, GSTs, esterases, and superoxide dismutases (SODs). Specifically, the GSTs and SODs are a group of a multifunctional antioxidant enzymes that play an important role in mediating oxidative stress caused by reactive oxygen species (ROS) in insects[92–94]. Therefore, the *miR-986* may be involved in the posttranslational regulation of genes that alleviate oxidative stress induced by DDT insecticide exposures. The putative targeted transcripts of *miR-986-5p* include *Cyp6g1* and *Cyp6g2*. However, the RT-qPCR performed here, as well as prior RNA-seq results [17], indicate that the expression levels of putative targets *Cyp6g1* and *Cyp6g2* were not decreased. In contrast, both *Cyp6g1* and *Cyp6g2* were significantly up-regulated in *91-R*, suggesting *miR-986-5p* may have a transcript stabilizing or enhancing effect, as was demonstrated previously [95,96]. Alternatively, algorithms used for *in silico* prediction of miRNA-transcript target interactions can produce variable results depending on the input database and models that are applied [97,98]. Regardless, *in vivo* or *in vitro* validation of these assumptions will be required.

## Conclusion

Analyses conducted in this study focused on differentially expressed miRNAs that were predicted to regulate transcript levels of both phase I, II and III detoxification genes previously shown to be associated with the DDT resistance phenotype. Cytochrome P450s that are involved in many cellular processes, including xenobiotic detoxification, have been studied [99] for associations between miRNA levels and corresponding putatively targeted P450 transcripts. For example, a negative relationship was shown between up-regulated miRNAs *miR-8534-5p* and *miR-375-5p* and their respective predicted targeted cytochrome P450s, *Cyp6b6* and Cyp4g15, in chlorantraniliprole-resistant strains of *Plutella xylostella* [44]. Several miRNAs down-regulated in deltamethrin-resistant mosquitoes played a role in pyrethroid resistance through the reduced targeting of *Cyp325bg3*, *Cyp6n23*, and *Cyp9j35* [47,48,50], while the miRNAs (*miR-155*, *miR-216b*, *miR-499*) modulate the abundance of *Cyp561d2* transcripts in response to fipronil exposure [100]. Additionally, *miR-285* and *miR-278* differentially regulate *Cyp6n23* and *Cyp6ag11* in pyrethroid resistant compared to susceptible *Culex pipiens* [48,49].

Thus, our implication of down-regulated *miR-311-3p*, *miR-312-3p*, and *miR-313-3p* with the corresponding constitutive up-regulation of *in silico* predicted targets *Cyp6a8*, *Cyp4s3*, *Cyp4ae1*, Cyp6g2, *Cyp6t3*, *Cyp6v1*, *Cyp18a1*, *Cyp49a1*, *Cyp303a1*, *Cyp309a2*, *Cyp313a4* in *91-R* may provide yet another example of a miRNA-mediated posttranscriptional modification that contributes to an insecticide resistance trait. Additionally, 4 out of 10 miRNAs predicted to be differentially expressed between *91-R* and *91-C* (*miR-986-5p*, *miR-995-3p*, *miR-312-3p*, *miR-2a-3p*) were also predicted to interact with and impact the transcript level of multidrug resistance-associated protein B7 (*ABC-B7)*. The ABC-B subfamily member, *MDR49*, had been previously been reported to show significant levels of differential expression between *91-R* and the DDT-susceptible strain *Canton-S* [12] but not between *91-R* and *91-C* [12,17]. It was subsequently shown that the *91-R*-derived *91-R-MDR49B* allele provided increased DDT tolerance via transgenic expression in susceptible *D*. *melanogaster*, implicating structural, as opposed to dosage, effects on the DDT resistance trait [14]. Thus, one could hypothesize that *ABC-B7* might be regulated by one or multiple miRNAs that are differentially expressed between *91-R and 91-C*. In addition to further empirical study, however, it should also be kept in mind that multiple different mutational mechanisms can contribute to DDT resistance in *91-R*.

In this study, we identified a set of differentially expressed known miRNAs and several novel miRNAs from DDT-resistant *91-R* and -susceptible *91-C* strains. Since *91-R* and *91-C* strains have a common initial origin (genetic background), and *91-R* has been selected for survival when exposed to chronic high levels of DDT for over six decades, changes between the strains are speculated to result from either the effects of random genetic drift or from selection. The experiments described here do not allow the differentiation of any impacts from drift versus selection, but the strong correlation between differential expression of miRNAs and their corresponding *in silico* predicted target transcripts suggests the potential for the involvement of posttranscriptional regulation of several detoxification genes.

Moreover, the experimental procedures do not allow the formulation that down-regulated miRNAs identified in the present study could lead directly to the overexpression of detoxification genes in the *91-R* strain, thus requiring further functional experiments in order to elucidate the mechanisms of miRNAs involvement in DDT resistance in *91-R*. As such, the overexpressed P450s putatively targeted by differentially-regulated miRNAs in *91-R* strain need to be further evaluated to identify the ones directly involved in the DDT resistance mechanism. Due to the polygenic nature of DDT resistance in *91-R* (see Introduction), however, this trait may be the result of interactions or additive/non-additive effects from several distinct genetic factors. This study, for the first time, suggests that these might include impacts mediated by miRNA posttranscriptional regulation. Our results provide valuable information for exploring the mechanisms of miRNAs involved in insecticide resistance and for understanding the evolution of post-transcriptional regulation in response to DDT pressure in *D*. *melanogaster*.

## Supporting information

S1 Fig Size distribution of small RNAs from the *91-R* and *91-C* libraries. (TIF)

S2 Fig Scatter plot of differentially expressed miRNA in the *91-R* and *91-C* strains. Each plot represents a miRNA. The X- and Y-axis show the normalized read counts of miRNAs in the two strains respectively.
(TIF)

S1 Table The list of RT-qPCR primers. (XLSX)

S2 Table Known miRNAs identified in this research. (XLSX)

S3 Table Known miRNA precursors not identified in this research. (XLSX)

S4 Table Novel miRNAs predicted in this research. (XLSX)

S5 Table Differentially expressed miRNAs between *91-R* and *91-C*. (XLSX)

S6 Table Target prediction of differentially expressed miRNAs between *91-R* and *91-C* with three algorithms. (XLSX)

S7 Table Gene ontology analysis of target transcripts from differentially expressed miRNAs between *91-R* and *91-C*. (XLSX)

S8 Table The pathway annotation of target transcripts from known differentially expressed miRNAs. (XLSX)

S9 Table Target genes for novel miRNAs predicted by RNAhybrid in this research. (XLSX)
