# Supplementary figures and images for: Differentially expressed microRNAs associated with changes of transcript levels in detoxification pathways and DDT-resistance in the Drosophila melanogaster strain 91-R
Source: PLoS One. 2018 Apr 26;13(4):e0196518. doi: 10.1371/journal.pone.0196518 (PMC5919617; doi:10.1371/journal.pone.0196518)

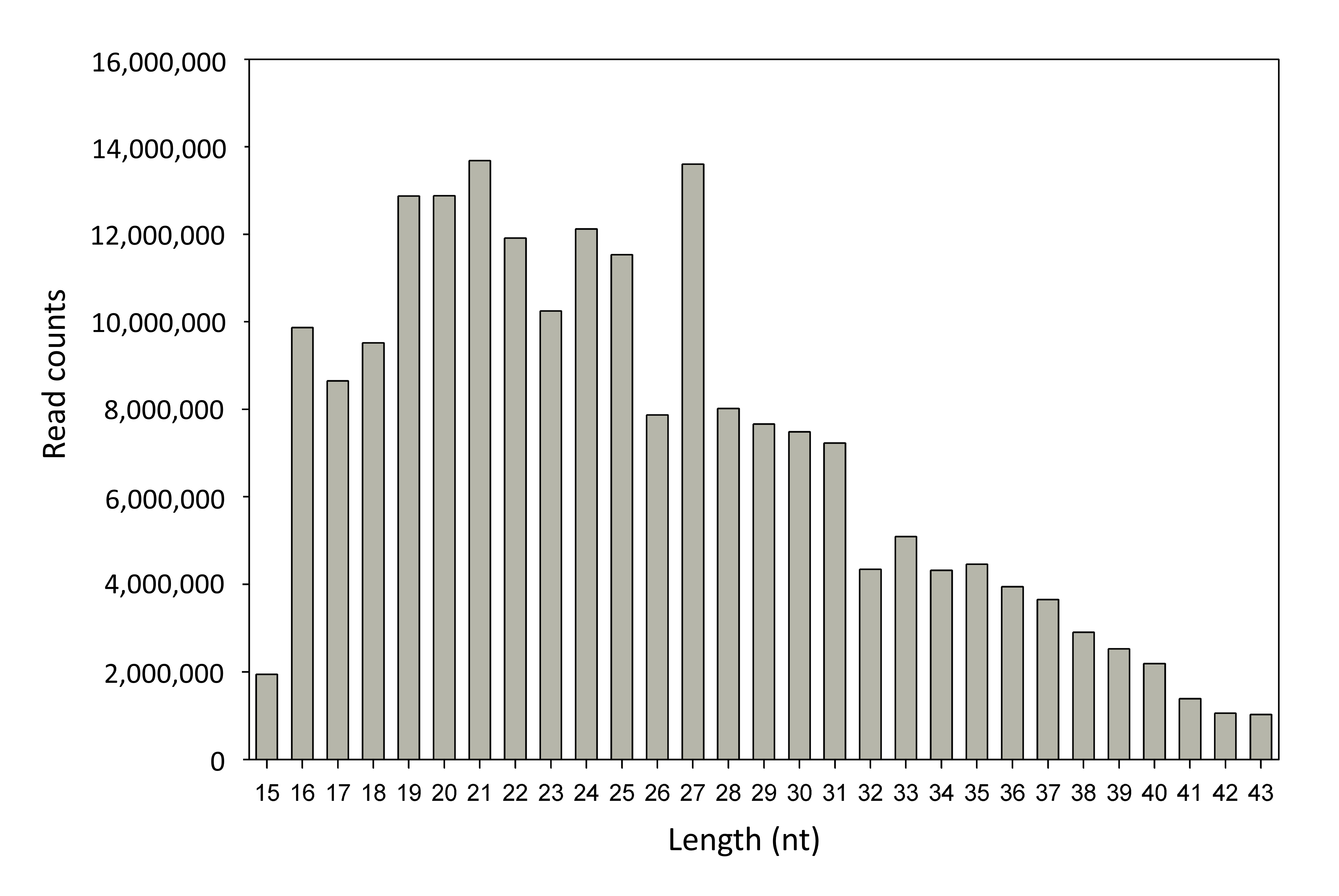

Supplement: S1 Fig — (TIF) [file pone.0196518.s001.tif]

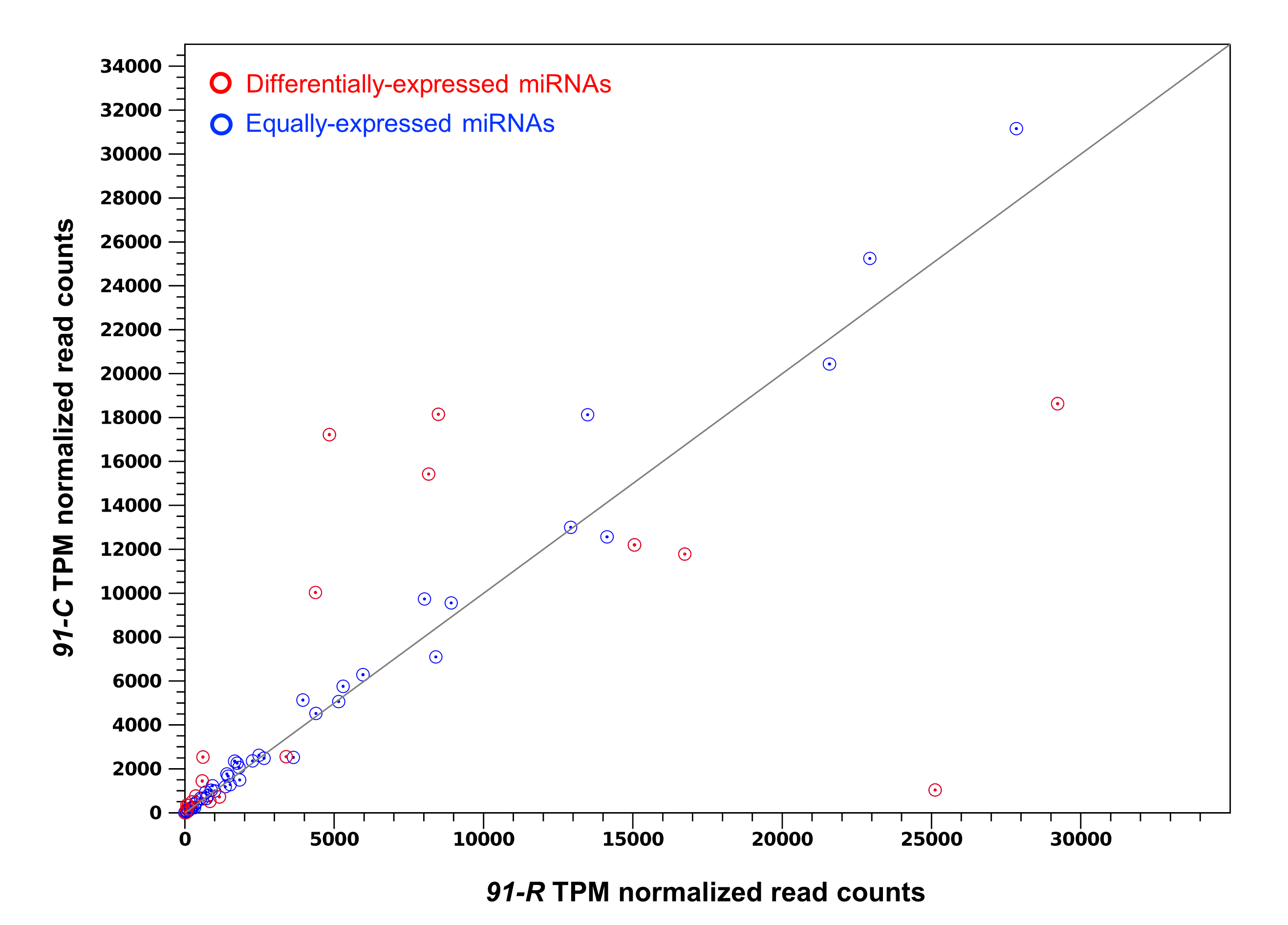

Supplement: S2 Fig — Each plot represents a miRNA. The X- and Y-axis show the normalized read counts of miRNAs in the two strains respectively. (TIF) [file pone.0196518.s002.tif]
